# Comparison of Intranasal Dexmedetomidine and Oral Midazolam for Premedication in Pediatric Dental Patients under General Anesthesia: A Randomised Clinical Trial

**DOI:** 10.1155/2020/5142913

**Published:** 2020-04-24

**Authors:** Li Wang, Lili Huang, Tiejun Zhang, Wei Peng

**Affiliations:** ^1^The State Key Laboratory Breeding Base of Basic Science of Stomatology (Hubei-MOST) and Key Laoratory of Oral Biomedicine Ministry of Education, Wuhan University, Wuhan, Hubei 430079, China; ^2^Department of Anesthesiology, School and Hospital of Stomatology, Wuhan University, Wuhan, Hubei 430079, China

## Abstract

The aim of this study was to compare the effects of preoperative intranasal dexmedetomidine and oral midazolam on preoperative sedation and postoperative agitation in pediatric dentistry. A total of 60 children (ASA grade I, aged 3–6 years) scheduled for elective pediatric dental treatment were randomly divided into the dexmedetomidine (DEX) and midazolam (MID) groups. Ramsay sedation score, parental separation anxiety scale, mask acceptance scale, pediatric anesthesia emergence delirium scale, and hemodynamic parameters were recorded. The Ramsay sedation scale and hemodynamic parameters of the children were observed and recorded immediately before administration and 10, 20, and 30 min after administration. A satisfactory mask acceptance scale rate was 93.33% in both MID and DEX groups, and there was no significant difference between the two groups (*p* > 0.05). The proportions of children that “successfully separated from their parents” were 93.33% (MID) and 96.67% (DEX). No significant difference was found between the two groups (*p* > 0.05). The incidence of agitation was 20% in the MID group and 0% in the DEX group, and the difference was statistically significant (*p* < 0.05). Intranasal dexmedetomidine and oral midazolam provided satisfactory sedation. No significant difference between the two groups was found in terms of parental separation anxiety and mask acceptance (*p* > 0.05). The incidence of postoperative pediatrics emergence delirium was significantly lower in the DEX group (*p* < 0.05).

## 1. Introduction

Young children often cannot tolerate dentistry in a routine clinical setting; thus, dentists frequently administer general anesthesia through an advanced behavioral management technique to provide quality dental care [1]. Pediatric patients frequently show uncooperativeness, fear, anxiety, and resistance when separated from their parents during venipuncture or when presented with an anesthetic mask. The administration of sedatives to a child before entering a surgical room is the most common way of reducing the child's distress and allows the child to undergo smooth anesthesia induction. Midazolam, which is anxiolytic, sedative, and hypnotic and exerts a compliant amnestic effect, has been widely used for premedication. However, it has some side effects, such as postoperative behavioral changes, cognitive impairment, paradoxical reactions, and respiratory depression. Additionally, midazolam is ineffective in preventing postoperative irritability compared with propofol, ketamine, dexmedetomidine, and fentanyl [2]. Dexmedetomidine is a highly selective *α*-2 agonist that produces sedative, anxiolytic, and analgesic effects without causing respiratory depression. Emergence agitation refers to thrashing, kicking, disorientation, inconsolable crying, hallucinations, and cognitive and memory impairment during the recovery period following the administration of general anesthesia [3]. Agitation can increase the amount of sedation and analgesics used and length of hospital stay and causes the appearance of associated complications [4]. Postoperative agitation in children undergoing different surgical procedures has been widely investigated. However, studies on postoperative agitation in children undergoing dental treatment under general anesthesia are few [5–7]. In this study, the effects of intranasal dexmedetomidine and oral midazolam were compared with those of preoperative sedation and postoperative agitation in 3–6-year-old children under dental treatment.

## 2. Materials and Methods

### 2.1. General Information

The study was approved by the Ethics Committee of the Hospital of Stomatology, Wuhan University ([2018] Ethical Review No. A [100]). Children who underwent full-mouth dental rehabilitation between January 2019 and August 2019 were enrolled. Before surgery, the significance and precautions of intranasal dexmedetomidine and oral midazolam were communicated to the parents of the children. The approval of the parents was then obtained, and informed consent was signed. The inclusion criteria were as follows: 3–6 years old, American Society of Anesthesiologists (ASA) grade I, and inability to cooperate in the pediatric dental clinic to treat children requiring general anesthesia. The exclusion criteria were as follows: patients with congenital diseases; allergy to dexmedetomidine, midazolam, or propofol; asthma; and patients with psychiatric and respiratory diseases. Finally, 60 children completed the study, which were divided into dexmedetomidine group on the basis of the random number table (DEX, *n* = 30) and midazolam group (MID, *n* = 30).

Through a computer-generated random list sealed and envelope technique, the 60 patients were equally divided into two groups in a random manner. Researchers not directly participating in patient care prepared the infusions, whereas anesthesiologists, dentists, and patients were unaware of the distribution of drugs and the groups.

### 2.2. Preoperative Administration and Anesthesia

All children were fasted for 8 h and deprived of water for 2 h before surgery. Under parental care, they entered the preoperative preparation room 30 min before surgery. In the DEX group, 2 *μ*g/kg dexmedetomidine hydrochloride injection was administered intranasally 30 min before the induction of anesthesia. In the MID, 0.5 mg/kg midazolam was administered orally 30 min before the induction of anesthesia. The dose of dexmedetomidine and midazolam was determined according to child weight. Dexmedetomidine was instilled into the nose, and midazolam was diluted with 5 mL of 50% glucose and administered orally. The two drugs were prepared by one of the investigators not directly involved in the care of the patient. The dental surgeon, anesthetist, and patients were unaware of which drug had been given. The heart rate (HR), oxygen saturation (SpO_2_) after administration, and respiratory rate (RR) of each child were monitored. At 10 min (T1), 20 min (T2), and 30 min (T3) after administration, the HR, RR, and SpO_2_ of each child were recorded by the anesthesiologist. At 30 min after medication, each child was transferred to a surgery room and presented with a sevoflurane mask for inhalation. Intravenous access was established after the eyelash reflex disappeared. Anesthesia induction was performed with intravenous 0.2 *μ*g/kg *sufentanil*, 2 mg/kg propofol, and 2 mg/kg cisatracurium besylate. Endotracheal intubation and mechanical ventilation were applied after successful induction. During the operation, propofol and remifentanil were pumped continuously and intravenously to maintain anesthesia, and cisatracurium was given intermittently to maintain muscle relaxation. Muscular relaxation was maintained with cisatracurium besylate. After anesthesia administration was started, appropriate surgery was performed on all the patients by the same surgeon. The dental procedure performed under general anesthesia in the introduction included root canal treatment, pulpotomy, and tooth extraction. Drug pump injection was stopped after the operation, and the patient was admitted into the anesthesia recovery room after oral suction and the children were removed in accordance with the conditions for tracheal catheter removal.

### 2.3. Observation Indicators

One of the investigators was not directly involved in the care of the patient and rated the study variables.

#### 2.3.1. Ramsay Sedation Score (RSS)

The scoring criteria were as follows: (1) patient shows anxiety and restlessness; (2) patient is cooperative, oriented, and quiet; (3) patient is responsive to instructions; (4) patient shows somnolence and responsive to the tapping of the glabella or to loud auditory stimuli; (5) patient shows somnolence and unresponsive to the tapping of the glabella or to loud auditory stimuli; and (6) patient shows somnolence without any response. RSSs were recorded immediately before and at 10, 20, and 30 min after dosing.

#### 2.3.2. Parental Separation Anxiety Scale (PSAS)

Anxiety score was determined when the child was separated from the parents according to four levels: (1) easy to separate; (2) sobbing but easy to cease; (3) crying loudly and difficult to stop but without ho+lding the parents and not letting them go; and (4) crying loudly and holding the parents and not willing to let them go. PSAS scores of 1 and 2 were considered “successful separation from parents.” The number of children that “successfully separated from their parents” was recorded in both groups.

#### 2.3.3. Mask Acceptance Scale (MAS)

The child's acceptance of the mask presented by the anesthesiologist was rated on four scales: 1 point, very good (not afraid, cooperative, easy to accept the mask); 2 points, good (slight fear of mask, easy to comfort); 3 points, moderate (moderate fear of mask, difficult to calm through comfort); and 4 points: poor (afraid, crying or struggling). In this study, both “score 1” and “score 2” were considered “satisfactory” mask reception behavior, and the number of children with “satisfactory” scores was recorded separately in both groups.

### 2.4. Statistical Analyses

Before the study, a power analysis suggested that a sample size of 25 patients in each group is required for the detection of a 30% difference in emergence delirium between DEX and control group at a beta level of 0.2 (80% power) and alpha level of 0.05 [8]. We rounded the groups to 60 patients. The results were presented as mean ± standard deviation for quantitative variables and summarized as absolute frequencies and percentages for categorical variables. Categorical variables were compared using the chi-square test or Fisher's exact test when more than 20% of the cells with an expected count of <5 were observed. Quantitative variables were compared with the Student's *t*-test. Variations in hemodynamic variables, including HR, RR, and SpO_2_, from the baseline among the groups were analyzed by repeated measures ANOVA. Statistical analysis was carried out using the SPSS software version 20. *p* values of ≤0.05 were considered statistically significant.

## 3. Results

### 3.1. Patients

**Figure 1:**
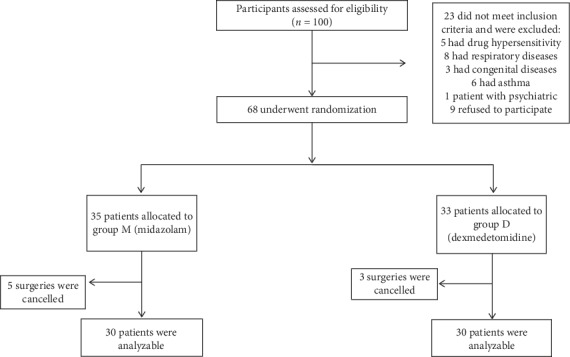
The flow diagram of the study.

100 participants were assessed for eligibility, 23 of these patients did not meet the inclusion criteria and were excluded, and 9 patients refused to participate. Then 68 patients were randomly assigned to two groups (M, *n* = 35 and D, *n* = 33) by use of a computer- generated randomization list. However, there were 5 and 3 patients in the midazolam and dexmedetomidine group who have been ruled out due to the surgeries cancelled, respectively. 60 patients completed the analysis, with 30 subjects in each treatment group, as illustrated in the flow diagram (Figure 1).

No significant difference was observed between the two groups in demographic variables, such as age, weight, gender composition, duration of surgery, and duration of anesthesia (*p* > 0.05), as shown in Table 1.

**Figure 2:**
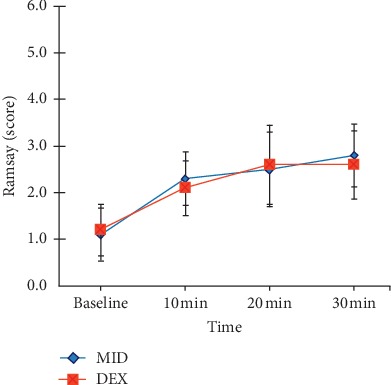
Ramsay scores (mean ± SD) for the two treatment groups (group M and group D). ^*∗*^*p* < 0.05.

Before medication, the difference in RSS between the two groups presented no statistically significant difference at 10, 20, and 30 min. Both groups had RSSs of ≥2, with no significant difference (*p* > 0.05), as shown in Figure 2.

**Figure 3:**
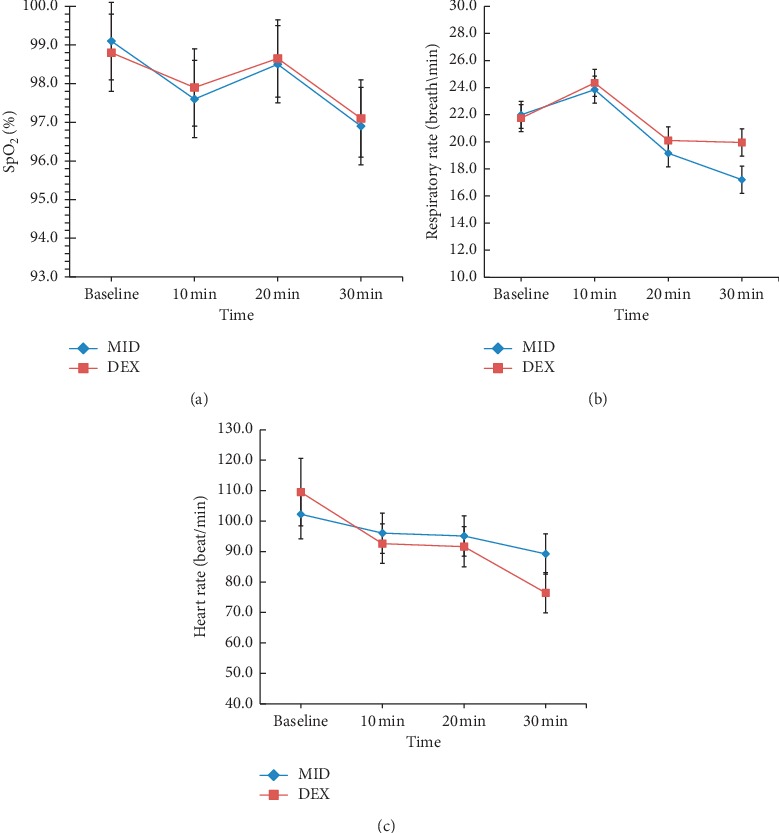
Mean heart rate, respiration rate, and SpO_2_ levels of the groups during the premedication period. Abbreviations: DEX: dexmedetomidine; MID: midazolam; SpO_2_: peripheral capillary oxygen saturation.

No child showed SpO_2_ of <95% during the dosing observation period. The mean HR, SpO_2_, and RR in both groups before and at 10, 20, and 30 min after dosing were not significantly different (*p* > 0.05, Figure 3).

MAS scores of “1” and “2” were considered “satisfactory” mask acceptance. A total of 28 children in the MID group received “1 point” and “2 points,” accounting for 93.33% of the total number of children in this group; 28 children in the DEX group also received “1 point” and “2 points,” and no significant difference was found between the two groups (*p*=1.00).

PSAS score analysis of the two groups of children showed 28 children with PSAS scores of “1 point” and “2 points” in the MID group, accounting for 93.33% of the total, and 29 children in the DEX group, accounting for 96.67% of the total. Most of the children in the two groups “successfully separated from their parents.” The data were compared between the two groups, and the difference was not statistically significant (*p*=0.95).

In the postanesthesia care unit, six children in the MID group with a total PAEDS score of ≥10 were considered agitated. No child in the DEX group showed a total PAEDS score of ≥10. The number of children experiencing agitation was significantly lower than that in the MID group, and the difference was statistically significant (*p*=0.01, Table 2).

## 4. Discussion

Dental diseases are common in children, and surgery is needed for the administration of general anesthesia. However, pediatric patients show anxiety and crying behavior because of numerous factors, such as separation from their parents and environmental changes before surgery. Some children refuse to enter the operating room and do not cooperate with anesthesia induction. Therefore, the administration of safe and effective preoperative sedatives is necessary. The preoperative administration of sedatives can reduce anxiety, psychological trauma, and anesthesia induction.

Midazolam is a common premedication in children and presents a rapid onset, short duration, and ability to provide reliable sedation and anxiolysis [9]. However, midazolam also has disadvantages, such as bitter taste, cognitive dysfunction, staged behavioral abnormalities, hiccups, and respiratory depression. Dexmedetomidine is increasingly used for premedication in children and is a novel *α*_2_ agonist that exerts sedative, analgesic, and anxiolytic effects in a dose-dependent manner [10]. It is effective and safe for preoperative sedation in children, has some analgesic effects, and does not cause respiratory depression [11].

The most common routes of midazolam administration are intravenous, intramuscular, oral, rectal, and intranasal. Dexmedetomidine can be administered intravenously, orally, intranasally, and intramuscularly [12]. Numerous studies have investigated the route and dosage of DEX. However, research on the optimal route and dose for DEX is still underway. Yuen et al. showed that 62% of children undergoing surgery have a satisfactory sedative effect with 1 *μ*g/kg dexmedetomidine nose drops before surgery [13]. Li et al. used 1.0 *μ*g/kg dexmedetomidine nasal drops 45–60 min before the induction of pediatric anesthesia, which was equally effective compared with 0.2 mg/kg midazolam nasal drops [14]. The antianxiety property was effective; however, the sedation satisfaction rate during the induction of anesthesia was lower than that of the midazolam group, suggesting that 1.0 *μ*g/kg dexmedetomidine may not be sufficient to achieve the desired depth of sedation.

In many studies, 2.0 *μ*g/kg dexmedetomidine was used for sedation before surgery. Talon et al. administered 2.0 *μ*g/kg dexmedetomidine nose drops for improved sedation [15]. Yuen et al. recommended 2.0 *μ*g/kg dexmedetomidine nasal drops before the induction of pediatric anesthesia in children aged 5–8 years; the nasal drops showed enhanced sedative effect without increasing the incidence of adverse reactions [16]. Simons et al. found that intranasal instillation is an effective preoperative route for pediatric patients [17]. It is relatively easy and noninvasive and has high bioavailability. The intranasal bioavailability of dexmedetomidine is 65% (35%–93%) and the oral bioavailability of dexmedetomidine is approximately 16% [18]. Dexmedetomidine does not contain preservatives and causes evident irritation upon nasal administration. The operation is simple, and the effect is rapid. Therefore, in this study, we used 0.5 mg/kg oral midazolam and 2 *μ*g/kg intranasal dexmedetomidine.

In this study, we assessed the depth of sedation in children through RSS, and we found that an RSS of 2 or 3 is the appropriate depth of sedation, during which a child is cooperative, conscious, oriented, and quiet. Jun et al. found that the oral administration of 2 *μ*g/kg dexmedetomidine and 0.5 mg/kg midazolam provides satisfactory mask acceptance and eases separation from parents 30 min before surgery [19]. Our study is consistent with the conclusions of the study by Jun et al. The RSSs in the two groups showed sedation scores of 2 to 3, and satisfactory results were achieved in terms of separation from parents and mask acceptance. Faritus et al. found that children that received preoperative oral administration of 0.5 mg/kg midazolam or 2 *μ*g/kg dexmedetomidine can be easily separated from their parents and received an anesthesia mask without significant hemodynamic changes during congenital heart disease surgery [20]. Similarly, no significant difference in changes in blood pressure and HR were found between the two groups in our study. Furthermore, preoperative intranasal dexmedetomidine or midazolam had no significant effect on a child's HR, respiration, and finger SpO_2_. In fact whether dexmedetomidine causes hypotension, bradycardia, and hypoxia depends on the dose and route of administration. Although HR was significantly lower in the dexmedetomidine group 30 min after dosing, the change in HR remained within the normal range and was not significantly different from that in the midazolam group in our study.

The preoperative anxiety of patients must be reduced not only for the improvement of preoperative cooperations but also for the prognosis of patients. Sheta et al. reported that incidence of agitation after the administration of 1 *μ*g/kg intranasal dexmedetomidine as premedication was lower than that obtained after 0.2 mg/kg intranasal midazolam in children undergoing full-mouth dental rehabilitation [21]. Jannu et al. compared the effect of administering 4 *μ*g/kg of oral dexmedetomidine as premedication with that of administering 0.75 mg/kg of oral midazolam and observed a low incidence of agitation in children premedicated with dexmedetomidine [22]. Jannu et al. reported that preoperative oral administration of 4 *μ*g/kg dexmedetomidine or oral administration of 0.75 mg/kg midazolam decreases the incidence of agitation in children aged 1–7 years [23]. Prabhu and Mehandale compared preoperative oral administration of 4 *μ*g/kg dexmedetomidine and oral administration of 0.5 mg/kg midazolam and concluded that the incidence of intranasal agitation with dexmedetomidine increased [24]. In contrast to these studies, our study showed a 0% incidence of agitation in the dexmedetomidine group and 20% in the midazolam group. The results revealed that the incidence of agitation was significantly lower in the dexmedetomidine group than in the midazolam group.

This clinical study had some limitations. First, the intravenous formulation of DEX was used because an intranasal preparation of DEX was not available. Second, although increasing clinical evidence shows that dexmedetomidine can be safely used in pediatric anesthesia, relevant authorities in various countries, including the FDA, have not yet approved its use for pediatric anesthesia. Thus, it remains an off-label medication. Regardless of these limitations, studies with large sample sizes are needed to determine the optimum doses of DEX and evaluate its safety and efficacy for the pediatric population.

## 5. Conclusion

In 3–6-year-old children undergoing oral therapy under general anesthesia, 2 *μ*g/kg preoperative intranasal dexmedetomidine and 0.5 mg/kg midazolam provided a satisfactory level of sedation. Most of the children were easily separated from their parents and presented good cooperation when presented with an anesthetic mask. Moreover, the incidence of postoperative agitation was lower in the dexmedetomidine group than in the midazolam group.

## Figures and Tables

**Table 1:** Comparison of demographic information, duration of operation, and duration of anesthesia between the groups.

	DEX group	MID group	*p* value
	*n* = 30	*n* = 30	
Age (years)	4.56 ± 0.59	4.79 ± 0.48	0.65
Weight (kg)	15.12 ± 2.14	14.87 ± 1.56	0.72
Gender (male/female)	16 : 14	15 : 15	0.49
Duration of operation (h)	2.59 ± 0.65	2.34 ± 0.71	0.28
Duration of anesthesia (h)	2.85 ± 0.49	2.62 ± 0.62	0.37

Note: data are expressed as mean ± SD or the number of children. Significant differences are at *p* < 0.05. Abbreviations:DEX: dexmedetomidine; MID: midazolam.

**Table 2:** Comparison of the groups in terms of preoperative cooperation and postoperative agitation in two groups (*n* = 30).

	MID group	DEX group	*X* ^2^/*p* value
	*n* (%)	*n* (/%)	
*Mask acceptance*			0.00/1.00
Satisfactory	28 (93.33%)	28 (93.33%)	
Unsatisfactory	2 (6.67%)	2 (6.67%)	

*Successful parental separation*			0.08/0.95
Yes	28 (93.33%)	29 (96.67%)	
No	2 (6.67%)	1 (3.33%)	

*Emergence delirium*			4.95/0.01^*∗*^
Present	6 (20.00%)	0 (0%)	
Absent	24 (80.00%)	30 (100%)	

Note: values in number (%). ^*∗*^Significant differences between groups at the 0.05 level. Abbreviations: DEX: dexmedetomidine; MID: midazolam.

## Data Availability

The data used to support the findings of this study are available from the corresponding author upon request.
